# Yoonia algicola sp. nov., Yoonia rhodophyticola sp. nov. and Yoonia phaeophyticola sp. nov., isolated from marine algae

**DOI:** 10.1099/ijsem.0.006545

**Published:** 2024-10-16

**Authors:** Dong Min Han, Ji Hoon Jeon, Myeong Seo Jin, Dae Gyu Choi, Jeong Min Kim, Hülya Bayburt, Byeong Jun Choi, Che Ok Jeon

**Affiliations:** 1 Department of Life Science, Chung-Ang University, Seoul 06974, Republic of Korea

**Keywords:** marine algae, new taxa, *Yoonia algicola*, *Yoonia phaeophyticola*, *Yoonia rhodophyticola*

## Abstract

Three Gram-stain-negative, strictly aerobic, non-motile, oxidase- and catalase-positive, short-rod-shaped bacteria, designated as strains G8-12^T^, SS1-5^T^ and BS5-3^T^, were isolated from marine algae in South Korea. Strain G8-12^T^ exhibited optimal growth at 20–25 °C, pH 8.0 and 2.0–2.5% (w/v) NaCl, while strains SS1-5^T^ and BS5-3^T^ grew optimally at 25 °C, pH 7.0 and 1.5% NaCl. All strains contained ubiquinone-10 as the sole respiratory quinone, with phosphatidylglycerol and phosphatidylcholine as major polar lipids, and C_18 : 1_* ω*7*c* and C_16 : 0_ as major fatty acids (>5 %); C_18 : 1_* ω*7*c* 11-methyl and C_18 : 1_ 2-OH were additionally identified as major fatty acids in strain SS1-5^T^. The genomic DNA G+C contents were 57.0, 58.3 and 56.4% for strains G8-12^T^, SS1-5^T^ and BS5-3^T^, respectively. Strains G8-12^T^, SS1-5^T^ and BS5-3^T^ exhibited less than 74.8% average nucleotide identity (ANI) and 19.7% digital DNA–DNA hybridization (dDDH) values with each other, indicating that they represent different species. Phylogenetic analyses based on both 16S rRNA gene and genome sequences revealed that strains G8-12^T^, SS1-5^T^ and BS5-3^T^ form distinct phylogenetic lineages within the genus *Yoonia*. Relative to other closely related *Yoonia* species, these strains exhibited ANI and dDDH values below 83.5 and 26.9%, respectively, suggesting that they constitute novel species within the genus *Yoonia*. Based on their phenotypic, chemotaxonomic and phylogenetic characteristics, strains G8-12^T^, SS1-5^T^ and BS5-3^T^ represent three novel species of the genus *Yoonia*, for which the names *Yoonia algicola* sp. nov. (G8-12^T^=KACC 22753^T^=JCM 35790^T^), *Yoonia rhodophyticola* sp. nov. (SS1-5^T^=KACC 22649^T^=JCM 35753^T^) and *Yoonia phaeophyticola* sp. nov. (BS5-3^T^=KACC 22648^T^=JCM 35751^T^) are proposed, respectively.

## Introduction

The genus *Yoonia*, a member of the family *Rhodobacteraceae* within the phylum *Pseudomonadota*, was initially established by Wirth and Whitman through the reclassification of certain species from the genus *Loktanella*, with *Yoonia vestfoldensis* as the type species [1]. As of May 2024, this genus includes nine species with validly published names (https://lpsn.dsmz.de/genus/yoonia). Members of the genus *Yoonia* have been primarily isolated from marine environments, including microbial mats in Antarctic lakes [2], seawater [3,8] and marine sediment [9,10]. These bacteria are Gram stain negative, non-spore forming, aerobic, generally coccoid, ovoid or rod-shaped and usually non-flagellated except for *Yoonia tamlensis*. Additionally, they exhibit catalase and oxidase activities but lack the ability to reduce nitrate [2,10]. Cells of the genus *Yoonia* contain ubiquinone-10 (Q-10) as the major respiratory quinone and C_18 : 1_* ω*7*c* and C_16 : 0_ as major cellular fatty acids. Their DNA G+C content ranges from 53.4 to 61.8%, and the major polar lipids detected in these cells include phosphatidylcholine (PC), phosphatidylglycerol (PG), diphosphatidylglycerol (DPG) and phosphatidylethanolamine (PE) [2,10]. During our research on the interactions between marine algae and bacteria, we isolated numerous novel bacteria from the phycosphere of marine algae [11,13]. In this study, we identified three potentially novel strains affiliated with the genus *Yoonia* from marine algae and characterized their taxonomic properties using a polyphasic approach. Additionally, members of the genus *Yoonia* have been recognized as core taxa in the phycosphere of marine algae [14], suggesting potential symbiotic relationships. Therefore, we further investigated marine algal symbiosis-associated genes in the genomes of three strains.

## Strain isolation

Strains G8-12^T^, SS1-5^T^ and BS5-3^T^ were isolated from marine algae classified as *Pyropia* sp., *Besa catenata* and *Sargassum* sp., respectively, which were collected from the coasts of Gonghyeonjin (38° 21′ 22″ N 128° 30′ 43″ E), Sinsi island (35° 49′ 29″ N 126° 27′ 39″ E) and Byeonsan (35° 40′ 55″ N 126° 31′ 44″ E) in the Republic of Korea, respectively, as previously described [13]. Briefly, the collected marine algae were thoroughly washed with artificial seawater (ASW; 20.0 g NaCl, 2.9 g MgSO_4_, 4.53 g MgCl_2_∙6H_2_O, 0.64 g KCl and 1.75 g CaCl_2_∙2H_2_O per litre) via mechanical vortexing. Subsequently, the washed algae were mechanically homogenized using an Ultra-Turrax homogenizer (IKA, Germany) for 10 s and then serially diluted in ASW. Aliquots of each dilution were spread onto marine agar (MA; MBcell, South Korea) plates, which were then aerobically incubated at 25 °C until colonies became visible. Colonies grown on MA were subjected to PCR amplification using universal primers 27F (5′-AGA GTT TGA TCM TGG CTC AG-3′) and 1492R (5′-TAC GGY TAC CTT GTT ACG ACT T-3′) [13]. The PCR products were then digested with *Hae*III and *Hha*I and analysed via 2% agarose gel electrophoresis. PCR products displaying distinct fragment patterns were partially sequenced with the 340F universal primer (5′-CCT ACG GGA GGC AGC AG-3′) [13] at Macrogen (South Korea). The partial 16S rRNA sequences were compared with those of all type strains of validly published species on the EzBioCloud server (http://www.ezbiocloud.net/) [15]. Based on this comparison, the potential novel strains, G8-12^T^, SS1-5^T^ and BS5-3^T^, belonging to the genus *Yoonia*, were selected for further taxonomic characterization. These strains were routinely cultured aerobically on MA agar at 25 °C for 3 days and preserved at –80 °C in marine broth (MB; BD, USA) supplemented with 15% (v/v) glycerol.

## Phylogeny based on 16S rRNA gene sequences

The nearly complete 16S rRNA gene sequences of strains G8-12^T^ (1409 nucleotides), SS1-5^T^ (1399 nucleotides) and BS5-3^T^ (1406 nucleotides) were obtained by sequencing PCR amplicons (amplified with the 27F and 1492R primers) using the universal primers 340F, 518R (5′-ATT ACC GCG GCT GCT GG-3′) and 805F (5′-GAT TAG ATA CCC TGG TAG TC-3′) [12], after which the obtained sequences were assembled. Similarities among the 16S rRNA gene sequences of strains G8-12^T^, SS1-5^T^ and BS5-3^T^, as well as their closely related type strains, were calculated using EzBioCloud. The 16S rRNA gene sequences were aligned using the fast secondary-structure-aware Infernal aligner (ver. 1.1.4) [16], and phylogenetic trees were constructed using the neighbour-joining (NJ), maximum-likelihood (ML) and maximum-parsimony (MP) algorithms with bootstrap values (1000 replications) using the mega11 software [17]. The Kimura two-parameter model, nearest-neighbour-interchange heuristic search method and pairwise deletion options were applied to construct the NJ, MP and ML trees, respectively.

**Fig. 1.**
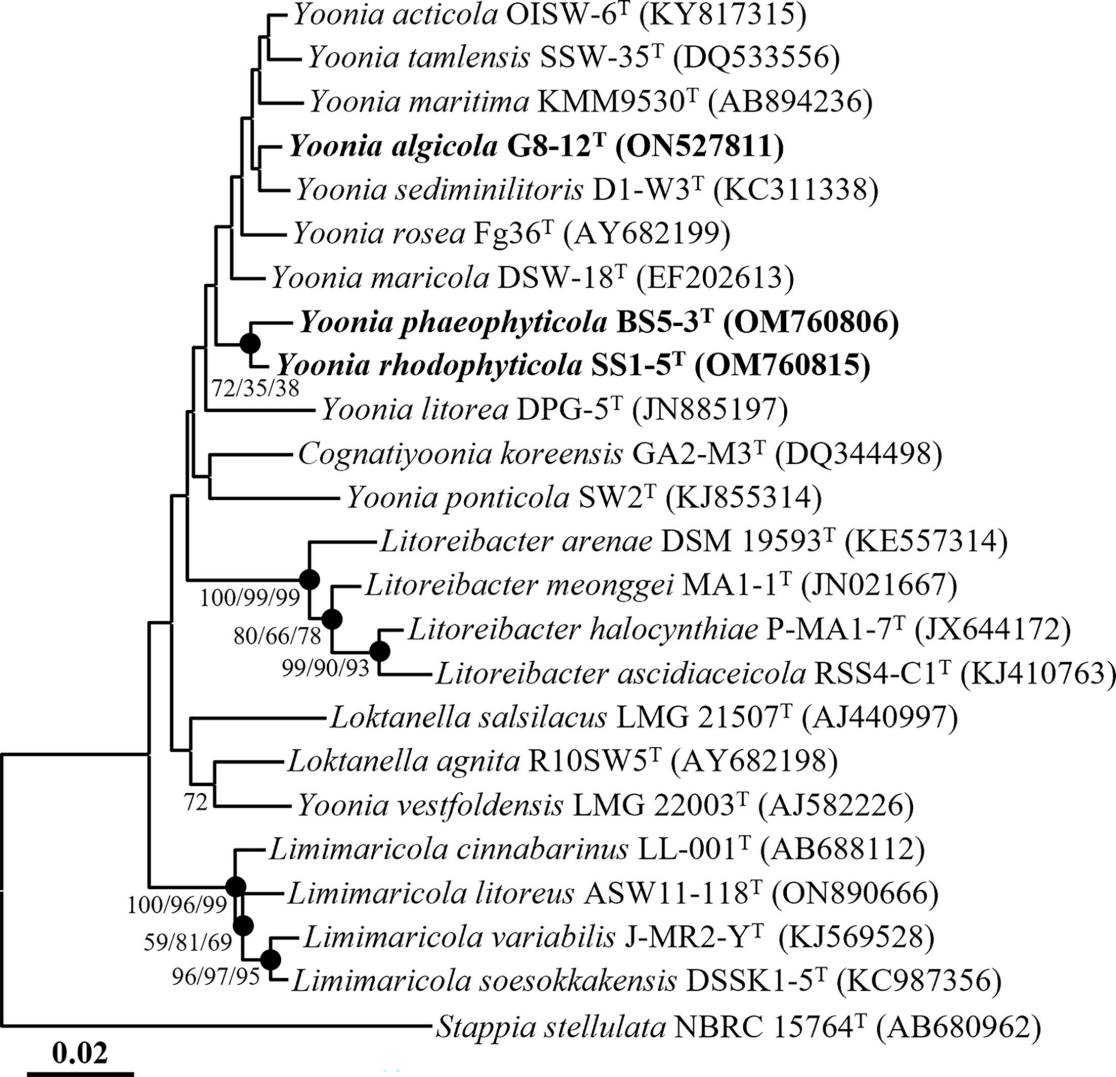
NJ phylogenetic tree based on 16S rRNA gene sequences, illustrating the phylogenetic relationships between strains G8-12^T^, SS1-5^T^ and BS5-3^T^ and their closely related taxa. Filled circles (●) indicate nodes that were also supported by ML and MP analyses. Bootstrap values are provided for nodes where at least one of the NJ, ML or MP algorithms yielded a value greater than 70%, based on 1000 replicates. *Stappia stellulata* NBRC 15764^T^ (AB680962) served as the outgroup. The scale bar represents 0.02 substitutions per nucleotide position.

Pairwise comparison of the 16S rRNA gene sequences revealed that strain G8-12^T^ was most closely related to *Yoonia rosea* Fg36^T^ (99.0%), *Yoonia sediminilitoris* D1-W3^T^ (98.9%) and *Yoonia acticola* OISW-6^T^ (98.8%); strain SS1-5^T^ was most closely related to *Yoonia maricola* DSW-18^T^ (98.3%), *Y. sediminilitoris* D1-W3^T^ (98.1%) and *Y. acticola* OISW-6^T^ (98.0%) and strain BS5-3^T^ was most closely related to *Y. maricola* DSW-18^T^ (98.4%), *Cognatiyoonia koreensis* DSM 17925^T^ (97.6%) and *Y. acticola* OISW-6^T^ (97.6%). Phylogenetic analysis based on 16S rRNA gene sequences using the NJ algorithm revealed that strains G8-12^T^, SS1-5^T^ and BS5-3^T^ formed distinct phylogenetic lineages within the genus *Yoonia* (Fig. 1). Phylogenetic trees constructed using the ML and MP algorithms further confirmed that strains G8-12^T^, SS1-5^T^ and BS5-3^T^ clustered with the members of the genus *Yoonia* (Fig. S1, available in the online Supplementary Material). These combined results from comparative and phylogenetic analyses based on 16S rRNA gene sequences suggest that strains G8-12^T^, SS1-5^T^ and BS5-3^T^ are members of the genus *Yoonia*. Based on the similarities and phylogenetic trees of the 16S rRNA sequences, *Y. maricola* KCTC 12863^T^, *Y. rosea* KCTC 22197^T^, *Y. sediminilitoris* KCTC 32383^T^ and *Y. vestfoldensis* KACC 13739^T^ (the type species of the genus *Yoonia*) were selected for comparisons of fatty acid compositions, as well as genomic and phenotypic characteristics.

## Insights into ecological distribution

The potential ecological habitat distribution of strains G8-12^T^, SS1-5^T^ and BS5-3^T^ was investigated by comparing their 16S rRNA gene sequences against metagenome-derived 16S rRNA data using the Integrated Microbial Next Generation Sequencing (IMNGS) platform, with a 99.0% sequence similarity threshold [18]. This analysis revealed that the 16S rRNA gene sequences of these strains matched metagenomic datasets from various environments, including marine, aquatic, sediment, oyster, coral, soil, plant, air, epibiont and gut microbiomes (Table S1). Notably, strains G8-12^T^, SS1-5^T^ and BS5-3^T^, particularly G8-12^T^, were most frequently identified in marine-related datasets, such as marine metagenomes, seawater metagenomes, salt lake metagenomes, beach sand and marine sediment metagenomes, indicating that marine environments are their primary habitats. However, the 16S rRNA sequences of these strains, particularly SS1-5^T^, were also commonly found in other environments, including air, plant, bioreactor and epibiont metagenomes, suggesting a broad ecological distribution.

## Integrated Microbial Next Generation Sequencing

IMNGS (platform, with a 99.0% sequence similarity threshold [18]. This analysis revealed that the 16S rRNA gene sequences of these strains matched metagenomic datasets from various environments, including marine, aquatic, sediment, oyster, coral, soil, plant, air, epibiont and gut microbiomes (Table S1). Notably, strains G8-12^T^, SS1-5^T^ and BS5-3^T^, particularly G8-12^T^, were most frequently identified in marine-related datasets, such as marine metagenomes, seawater metagenomes, salt lake metagenomes, beach sand and marine sediment metagenomes, indicating that marine environments are their primary habitats. However, the 16S rRNA sequences of these strains, particularly SS1-5^T^, were also commonly found in other environments, including air, plant, bioreactor and epibiont metagenomes, suggesting a broad ecological distribution.

## Whole-genome sequencing and genome-based phylogeny

The genomic DNA of strains G8-12^T^, SS1-5^T^ and BS5-3^T^ was extracted from cells cultured in MB using the Wizard Genomic DNA Purification Kit (Promega, USA), following the manufacturer’s instructions. The extracted genomic DNA was sequenced in-house using the Oxford Nanopore MinION (ONT, UK), and the resulting sequencing reads were *de novo* assembled using Flye (ver. 2.9.3) [19]. The quality of the assembled genomes was assessed using the CheckM2 software (ver. 1.0.2) [20], evaluating their completeness and contamination rates.

The *de novo* assembly of genomic sequencing reads from strains G8-12^T^, SS1-5^T^ and BS5-3^T^, with average genome coverages of ~103.0×, 146.0× and 72.0×, respectively, yielded complete genomes. Strain G8-12^T^ comprised two contigs, including a 3634 kb chromosome and an 11.5 kb plasmid; strain SS1-5^T^ comprised two contigs, including a 4132 kb chromosome and a 335.7 kb plasmid, and strain BS5-3^T^ comprised three contigs, including a 3901 kb chromosome and two plasmids of 64.5 and 16.6 kb (Table 1). The 16S rRNA gene sequences identified in the genomes of strains G8-12^T^, SS1-5^T^ and BS5-3^T^ were consistent with those obtained through PCR-based sequencing. The quality checks of the assembled genomes for strains G8-12^T^, SS1-5^T^ and BS5-3^T^ showed completeness rates of 99.97, 99.97 and 99.99% and contamination rates of 0.07, 0.10, and 0.0%, respectively, meeting the criteria for high-quality genomes (≥90% completeness, ≤10% contamination rate) [20]. All genomic sequencing data and assembled genome qualities of strains G8-12^T^, SS1-5^T^ and BS5-3^T^ satisfied the proposed minimal standards for the use of genome data for the taxonomy of prokaryotes [21].

**Table 1.** General genomic features of strains G8-12^T^, SS1-5^T^ and BS5-3^T^ and closely related type strains of the genus *Yoonia* Taxa: 1, strain G8-12^T^ (CP151762–3); 2, strain SS1-5^T^ (CP151764, CP151767); 3, strain BS5-3^T^ (CP150951–3); 4, *Y. maricola* DSM 29128^T^ (PGTY00000000); 5, *Y. rosea* DSM 29591^T^ (FTPR00000000); 6, *Y. sediminilitoris* DSM 29955^T^ (QBUD00000000); 7, *Y. Vestfoldensis* DSM 16212^T^ (ARNL00000000). The genomes of strains G8-12^T^, SS1-5^T^ and BS5-3^T^ were sequenced in this study.

Features*	1	2	3	4	5	6	7
Genome status† (no. of contigs)	C (2)	C (2)	C (3)	D (7)	D (5)	D (49)	D (45)
Total genome size (kb)	3645	4468	3982	3798	3514	4668	3722
G+C content (%)	57.0	58.3	56.4	56.2	57.7	57.2	61.8
No. of total genes	3687	4392	4036	3836	3543	4543	3792
No. of protein CDS	3460	4295	3981	3773	3483	4384	3700
No. of total RNA genes	52	44	55	46	48	50	60
No. of tRNA genes	43	38	46	40	42	44	48
No. of rRNA (16S, 23S, 5S) operons	2	1	2	1	1	1	3
No. of pseudogenes	175	53	45	17	12	109	32
No. of total CAZyme† genes	71	87	81	70	76	89	70
Glycoside hydrolases	19	38	26	24	24	36	24
Glycosyl transferases	42	36	37	34	41	33	34
Polysaccharide lyases	0	1	3	2	0	0	2
Auxiliary activities	5	4	6	4	6	10	4
Carbohydrate esterases	4	5	8	5	4	6	5
Carbohydrate-binding modules	1	3	1	1	1	4	1

* The genomic features were analyzedanalysed using the NCBI prokaryotic genome annotation pipeline (www.ncbi.nlm.nih.gov/genome/annotation_prok/).

† C, complete; D, draft; CAZyme, Carbohydrate-Active enZyme.

A phylogenomic analysis of strains G8-12^T^, SS1-5^T^ and BS5-3^T^ was conducted using the Genome Taxonomy Database Toolkit, based on the concatenated protein sequences of 120 ubiquitous single-copy marker genes (bac120 marker set) [22]. The alignment of these concatenated protein sequences and the subsequent phylogenomic ML tree reconstruction, including bootstrap values from 1000 replications, were performed using the mega11 software. Average nucleotide identity (ANI) and digital DNA–DNA hybridization (dDDH) values among strains G8-12^T^, SS1-5^T^ and BS5-3^T^, as well as other closely related type strains, were calculated using the Orthologous ANI Tool online (OAT, ver. 0.93.1; www.ezbiocloud.net/tools/orthoani) [23] and the Genome-to-Genome Distance Calculator (GGDC 3.0; https://ggdc.dsmz.de/ggdc.php) with formula 2 parameters [24], respectively.

**Fig. 2.**
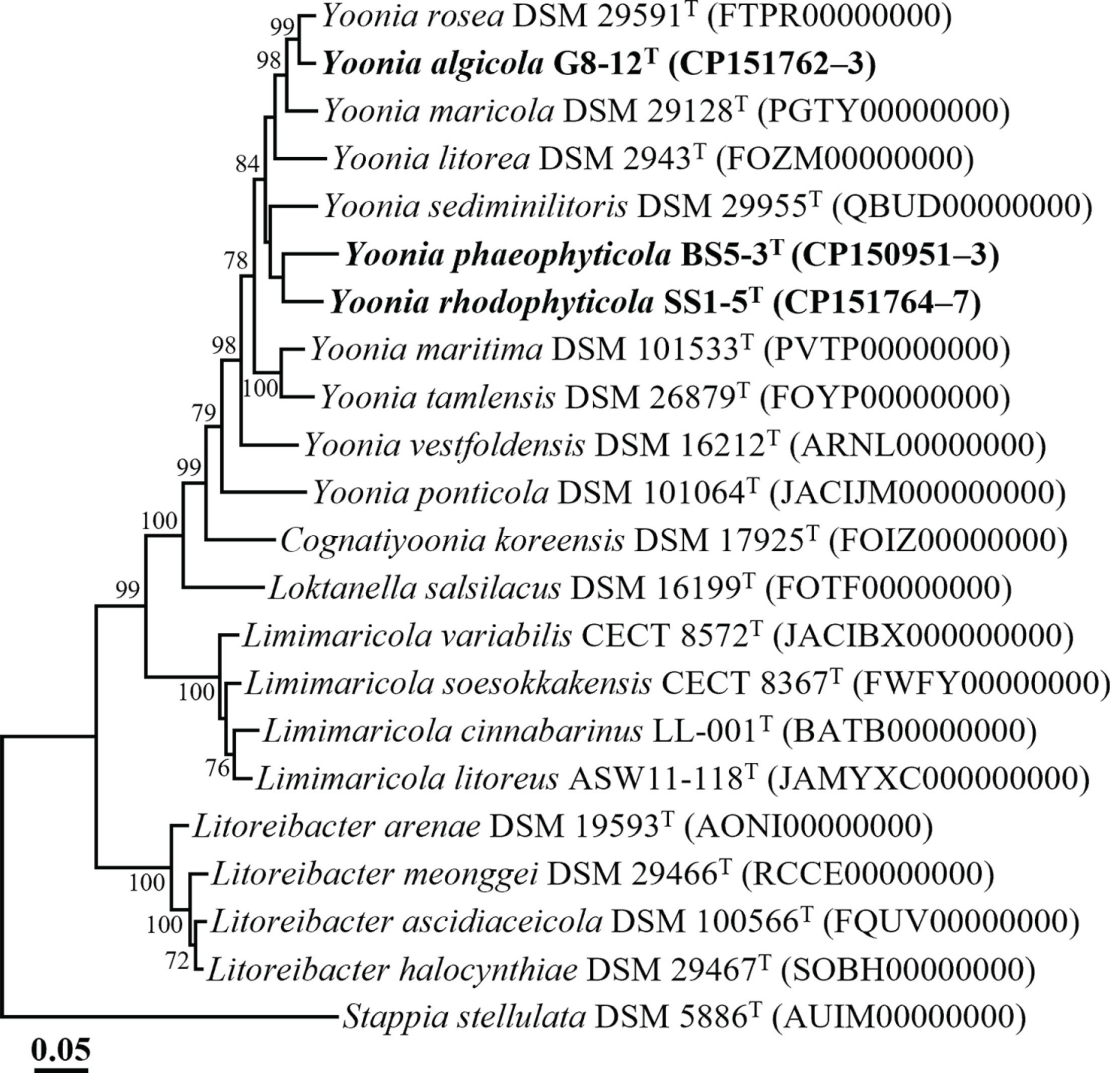
Phylogenomic tree depicting the phylogenetic relationships between strains G8-12^T^, SS1-5^T^ and BS5-3^T^ and closely related taxa, based on concatenated amino acid sequences from 120 single-copy marker genes (bac120 marker set), constructed using ML algorithms. Bootstrap values above 70% are indicated at the nodes. *S. stellulata* DSM 5886^T^ (AUIM00000000) was used as the outgroup. The scale bar represents 0.05 changes per amino acid position.

Genome-based phylogenomic tree analyses revealed that strains G8-12^T^, SS1-5^T^ and BS5-3^T^ formed distinct phylogenetic lineages within the genus *Yoonia* (Fig. 2), aligning with the conclusion from the 16S rRNA gene sequence analyses and confirming their classification within the genus *Yoonia*. Strains G8-12^T^, SS1-5^T^ and BS5-3^T^ exhibited ANI values below 74.8% and dDDH values below 19.7% among themselves and less than 83.5 and 26.9%, respectively, with their closely related type strains (Table S2). These values are significantly lower than the established prokaryotic species delineation thresholds (ANI, ~95%; dDDH, 70%) [21]. The results from the phylogenomic analysis and genome relatedness assessments strongly support the conclusion that strains G8-12^T^, SS1-5^T^ and BS5-3^T^ represent distinct novel species within the genus *Yoonia*.

## Genomic features and algal symbiosis-associated genes

The whole-genome sequences of strains G8-12^T^, SS1-5^T^ and BS5-3^T^ were submitted to GenBank and annotated using the NCBI Prokaryotic Genome Annotation Pipeline [25]. The genomes of strains G8-12^T^, SS1-5^T^ and BS5-3^T^ were predicted to contain a total of 3687, 4392 and 4036 genes, respectively, including 3460, 4295 and 3981 protein-coding sequences (CDS); 2, 1 and 2 rRNA operons (16S, 23S, 5S); and 43, 38 and 46 tRNA genes. The genomic features of the analysed strains, including genome size, total gene numbers, protein-coding genes and total tRNA gene numbers, were consistent with those of closely related *Yoonia* species (Table 1). Additionally, the G+C contents of strains G8-12^T^, SS1-5^T^ and BS5-3^T^ were 57.0, 58.3, and 56.4%, respectively, aligning with the DNA G+C content ranges of other *Yoonia* species [2,10].

Algae are primarily composed of polysaccharides, which are essential components of their extracellular matrices, cell walls and storage compounds. Therefore, the ability to degrade various algal polysaccharides is a significant trait among heterotrophic bacteria associated with marine algae [26]. To predict the algal polysaccharide-degrading abilities of strains G8-12^T^, SS1-5^T^ and BS5-3^T^, as well as closely related *Yoonia* strains, Carbohydrate-Active enZymes (CAZymes) were analysed by submitting the protein sequences from GenBank to the dbCAN3 meta server (https://bcb.unl.edu/dbCAN2/blast.php) [27]. The genomes of strains G8-12^T^, SS1-5^T^ and BS5-3^T^ were predicted to contain 71, 87 and 81 genes encoding various CAZymes, respectively, similar to those in other closely related *Yoonia* strains (Table 1), indicating a comparable capability to degrade algal polysaccharides. Strains G8-12^T^, SS1-5^T^ and BS5-3^T^ possess genes involved in the degradation of alginate, a major cell wall component of brown algae. Notably, strain BS5-3^T^, isolated from a brown alga, contains all the necessary genes for alginate degradation: PL5 (alginate lyase), PL7 (alginate lyase), PL15 (oligoalginate lyase) and GH3 (*β*-glucuronidase), indicating its ability to completely degrade alginate to d-mannuronate and l-guluronate. In contrast, strain SS1-5^T^ lacks PL5 and PL7, and strain G8-12^T^ lacks PL5, PL7 and PL15, indicating their incomplete ability to degrade alginate. This suggests that these strains may rely on cooperation with other bacteria that possess complementary alginate-degrading genes.

Symbiotic bacteria associated with marine algae play a crucial role in promoting algal growth by supplying beneficial compounds such as hormones, vitamins, siderophores and essential nutrients [28]. Bioinformatic analysis of the genomes of strains G8-12^T^, SS1-5^T^ and BS5-3^T^ revealed that all three strains possess complete glycolysis, gluconeogenesis and pentose phosphate pathways, as well as the complete TCA cycle, indicating their capability to efficiently produce energy from various carbon sources within the algal phycosphere. Additionally, all three strains harbour genes encoding enzymes responsible for synthesizing riboflavin from guanosine triphosphate (GTP) (*ribABDEH*) and ribulose 5-phosphate (*ribBEH*). Moreover, strains SS1-5^T^ and BS5-3^T^ possess genes for synthesizing thiamine from 4-amino-5-hydroxymethyl-2-methylpyrimidine (*thiDE* and thiamine monophosphate phosphohydrolase gene). Strains G8-12^T^ and BS5-3^T^ have the complete gene set necessary for the biosynthesis of cobalamin from glycine, including 5-aminolevulinate synthase, *hemBCD*, *cysG*, *cbiABCDEFGHJKLTP* and *cobAOSU* genes. In contrast, strain SS1-5^T^ lacks the *cbiADETP* and *hemB* genes. Notably, strain G8-12^T^ also possesses genes for the synthesis of pantothenate from pyruvate (*ilvCDM* and *panBCE*), folate from GTP (*folABCEKP* and *phoD*) and phenylacetate from l-phenylalanine (*katG* and *amiE*), a phytohormone that may enhance growth and stress tolerance in marine algae [28]. These findings suggest that strains G8-12^T^, SS1-5^T^ and BS5-3^T^ can supply essential nutrients, such as various vitamins, to their algal hosts, thereby facilitating symbiotic relationships with marine algae. Polyamines, such as putrescine, are known to enhance photosynthesis, cell proliferation and growth while providing protection against abiotic stresses in marine algae [28]. Strains G8-12^T^ and SS1-5^T^ possess genes (*rocF* and *speF*) that enable the conversion of arginine and ornithine into putrescine, whereas strain BS5-3^T^ possesses the *speB* gene for converting agmatine into putrescine. These capabilities likely support symbiosis with marine algae by enhancing stress response mechanisms.

## Morphological and physiological characteristics

The growth of strains G8-12^T^, SS1-5^T^ and BS5-3^T^ was tested on various agar media (all from MBcell), including MA, R2A agar, tryptic soy agar, nutrient agar and Luria–Bertani (LB) agar with 2% (w/v) NaCl, at 25 °C for 3 days. Growth was also examined at different temperatures (5–40 °C at 5 °C intervals) and pH values (4.0–10.0 at 1.0 pH unit intervals) on MA and in MB at 25 °C for 3 days. MB media with various pH levels were prepared using sodium citrate (pH 4.0–5.0), sodium phosphate (pH 6.0–8.0) and sodium carbonate-bicarbonate (pH 9.0–10.0) buffers, with pH adjustments after autoclaving if necessary. Salt tolerance was assessed in MB with different NaCl concentrations (0–5.0% at 0.5% intervals, w/v) at 25 °C for 3 days. Anaerobic growth was evaluated on MA after 21 days of incubation at 25 °C using the GasPak Plus system (BBL, USA).

All physiological and biochemical tests were conducted using cells grown on MA at 25 °C for 3 days. Cellular morphology and motility were examined with a phase-contrast microscope (Zeiss Axio Scope.A1; Carl Zeiss, Germany), and detailed morphology was observed using a transmission electron microscope (JEM-1010; JEOL, Japan) with 2 % (w/v) uranyl acetate (Sigma-Aldrich, USA) staining. Gram staining was performed using a Gram stain kit (bioMérieux, France) following the manufacturer’s instructions. Catalase and oxidase activities were tested using 3% (v/v) hydrogen peroxide (Junsei, Japan) and 1% (w/v) tetramethyl-*p*-phenylenediamine (Merck, USA), respectively. Hydrolysis of casein (1.0% skim milk, w/v), starch (1.0%), tyrosine (0.5%), Tween 20 (1.0%), Tween 80 (1.0%), and aesculin (0.1%) was assessed on MA at 25 °C for 3 days [29]. Additional biochemical features and enzymatic activities were evaluated using the API 20NE kit (bioMérieux) according to the manufacturer’s instructions.

Strains G8-12^T^, SS1-5^T^ and BS5-3^T^ exhibited robust growth on MA but did not grow on R2A agar, tryptic soy agar, nutrient agar or LB agar containing 2% NaCl. All strains were Gram stain negative, non-motile and strictly aerobic, as they did not grow under anaerobic conditions after 21 days. Morphologically, strains G8-12^T^, SS1-5^T^ and BS5-3^T^ appeared as short rods, measuring ~0.6–0.7×0.7–1.2 µm, 0.4–0.6×0.8–1.1 µm and 0.5–0.6×0.9–1.1 µm, respectively (Fig. S2). These strains shared many phenotypic, physiological and biochemical features with their closely related strains, such as Gram-staining results, non-motility, oxidase and catalase activities, nitrate reduction, the ability to hydrolyse Tween 20, Tween 80, aesculin and gelatin and indole production. However, there were some differences in growth range and the assimilation of certain carbon sources (Table 2).

**Table 2.** Comparison of phenotypic characteristics of strains G8-12^T^, SS1-5^T^ and BS5-3^T^ and closely related type strains of the genus *Yoonia* Taxa: 1, strain G8-12^T^ (this study); 2, strain SS1-5^T^ (this study); 3, strain BS5-3^T^ (this study); 4, *Y. maricola* KCTC 12863^T^ [3]; 5, *Y. rosea* KCTC 22197^T^ [8]; 6, *Y. sediminilitoris* KCTC 32383^T^ [9]; 7, *Y. vestfoldensis* KACC 13739^T^ [2]. All strains are positive for the following characteristics: activity* of oxidase, catalase, urease and *β*-galactosidase, and hydrolysis* of aesculin. All strains are negative for the following characteristics: Gram-staining, motility, indole production, nitrate reduction, glucose fermentation, hydrolysis of Tween 20, Tween 80, casein, starch and tyrosine and assimilation* of l-arabinose, d-mannose, d-mannitol, *N*-acetyl-glucosamine, capric acid, adipic acid, trisodium citrate and phenylacetic acid. Symbols: +, positive; –, negative; *W*, weak positive; na, not available.

Characteristics	1	2	3	4	5	6	7
Colony colour	Light yellow	Light yellow	Light yellow	Light orange	Pink	Greyish yellow	Pale pink
Growth range of							
Temperature (optimum, °C)	15–25(20–25)	15–25(25)	15–30(25)	4–34(na)	4–35(25)	10–35(25)	5–37(na)
pH (optimum)	6.0–9.0(8.0)	6.0–8.0(7.0)	6.0–8.0(7.0)	5.5–na(7.0–8.0)	6.0–10.0 (7.5–8.0)	5.5–na(7.0–8.0)	na
NaCl (%, w/v)	0.5–3.5	0.5–2.5	0.5–2.0	na–7.0	1.0–12.0	0–5.0	0–10.0
Arginine dihydrolase activity*	–	–	–	–	–	–	+
Gelatin hydrolysis*	+	+	+	*w*	+	*w*	–
Assimilation* of							
d-Glucose	+	–	–	–	–	*w*	+
d-Mannitol	–	–	–	–	–	–	+
d-Maltose	–	–	–	–	–	*w*	+
Potassium gluconate	–	–	–	–	–	*w*	–
Malic acid	–	–	–	+	–	–	+
Major polar lipids	PG, PC	PG, PC	PG, PC	PG, PC, DPG	PG, PC, DPG	PG, PC, PE, DPG	PG, PC, DPG

* These data were obtained from this study under the same conditions.

## Chemotaxonomic characteristics

The respiratory isoprenoid quinones of strains G8-12^T^, SS1-5^T^ and BS5-3^T^ were extracted from cells cultured in MB for 3 days at 25 °C, following the protocol described by Minnikin *et al*. [30]. These extracted quinones were analysed using an HPLC system (LC-20A; Shimadzu, Japan) equipped with a reversed-phase column (250×4.6 mm, Kromasil; Akzo Nobel, Netherlands) and a diode array detector (SPD-M20A; Shimadzu), with methanol–isopropanol (2 : 1, v/v) as the eluent at a flow rate of 1 ml min^−1^. The cellular fatty acids of strains G8-12^T^, SS1-5^T^ and BS5-3^T^, alongside four reference strains, were analysed from cells harvested during the exponential growth phase (OD_600_=0.7–0.8) in MB at 25 °C. Fatty acid methyl ester samples were prepared using the standard MIDI protocol (Sherlock Microbial Identification System, version 6.2B), involving saponification, methylation and extraction, and then analysed with a gas chromatograph (Hewlett Packard 6890, USA) and identified using the RTSBA6 database of the Microbial Identification System (Sherlock version 6.0B) [31]. The polar lipids of strains G8-12^T^, SS1-5^T^ and BS5-3^T^ and *Y. vestfoldensis* KACC 13739^T^ were analysed using two-dimensional thin-layer chromatography, as described by Minnikin *et al*. [32]. Various polar lipids were identified using 10% ethanolic molybdophosphoric acid (for total polar lipids), ninhydrin (for aminolipids), Dittmer-Lester (for phospholipids) and Dragendorff (for PC) reagents. The presence or absence of PG, PC and DPG in strains G8-12^T^, SS1-5^T^ and BS5-3^T^ was confirmed using standard polar lipid compounds from Sigma-Aldrich.

Q-10 was identified as the sole respiratory quinone in strains G8-12^T^, SS1-5^T^ and BS5-3^T^, consistent with other *Yoonia* species [3,9]. The major cellular fatty acids (>5% of the total) in all three strains included C_18 : 1_* ω*7*c* and C_16 : 0_, aligning with closely related *Yoonia* strains (Table S3). However, strain SS1-5^T^ also had C_18 : 1_* ω*7*c* 11-methyl and C_15 : 0_ 2-OH as major fatty acids. While the overall fatty acid profiles of strains G8-12^T^, SS1-5^T^ and BS5-3^T^ were similar to those of other *Yoonia* strains, there were differences in the proportions of certain fatty acids. For example, C_15 : 0_ 2-OH and C_10 : 0_ 3-OH, identified in other *Yoonia* strains, were present in strains G8-12^T^ and SS1-5^T^, respectively. PG and PC were identified as the major polar lipid components in all strains (G8-12^T^, SS1-5^T^ and BS5-3^T^). Strain G8-12^T^ also contained an unidentified aminolipid and two unidentified lipids, while strain SS1-5^T^ had an unidentified aminolipid and one unidentified lipid, and strain BS5-3^T^ contained an unidentified aminolipid (Fig. S3). The presence of PG and PC as major polar lipids in these strains aligns with other *Yoonia* species [3,10], including *Y. vestfoldensis*, the type species of the genus *Yoonia* (analysed in this study, Fig. S3). However, DPG, found in other *Yoonia* species, including *Y. vestfoldensis*, was not detected in these strains (Table 2).

## Taxonomic conclusion

The phylogenetic inference, genomic relatedness and phenotypic, biochemical and chemotaxonomic characteristics strongly support that strains G8-12^T^, SS1-5^T^ and BS5-3^T^ represent three different novel species of the genus *Yoonia*. Therefore, we propose the names *Yoonia algicola* sp. nov., *Yoonia rhodophyticola* sp. nov. and *Yoonia phaeophyticola* sp. nov. for these newly identified species, respectively.

## Description of *Yoonia algicola* sp. nov.

*Yoonia algicola* (al.gi′co.la. L. fem. n. *alga*, an alga; L. suffix. -*cola*, (from L. masc. or fem. n. *incola*), inhabitant, dweller; N.L. fem. n. *algicola*, an alga dweller).

Colonies on MA are light yellow, circular and convex. Cells are Gram-stain-negative, strictly aerobic and non-motile short rods. Growth occurs at 15–25 °C (optimum, 20–25 °C) and pH 6.0–9.0 (optimum, 8.0) and in the presence of 0.5–3.5% (w/v) NaCl (optimum, 2.0–2.5%). Oxidase and catalase activities are positive. Nitrate is not reduced to nitrite. Aesculin and gelatin are hydrolysed, but casein, l-tyrosine, Tween 20, Tween 80 and starch are not. Glucose fermentation and indole production are negative. *β*-Galactosidase and urease activities are positive, but arginine dihydrolase activity is negative. Assimilation of d-glucose is positive, but assimilation of d-mannose, *N*-acetylglucosamine, d-maltose, malic acid, trisodium citrate, l-arabinose, d-mannitol, capric acid, phenylacetic acid, potassium gluconate and adipic acid is negative. Q-10 is the sole respiratory quinone, and PG, PC, an identified amino lipid and two identified lipids are detected as major polar lipids. The major fatty acids (>5 % of the total fatty acids) are C_18 : 0_* ω*7*c* and C_16 : 0_.

The type strain is G8-12^T^ (=KACC 22753^T^=JCM 35790^T^), isolated from red marine algae (*Pyropia* sp.) collected from the coast of Gonghyeonjin, Republic of Korea. The genome size and DNA G+C content of the type strain are 3645 kb and 57.0% (calculated from the whole-genome sequence), respectively. The GenBank accession numbers for the 16S rRNA gene and genome sequences of strain G8-12^T^ are ON527811 and CP151762–CP151763, respectively.

## Description of *Yoonia rhodophyticola* sp. nov.

*Yoonia rhodophyticola* (rho.do.phy.ti′co.la. N.L. neut. pl. n. *Rhodophyta*, the division of the red algae; L. suffix. -*cola* (from L. masc. or fem. n. *incola*), inhabitant, dweller; N.L. fem. n. *rhodophyticola*, inhabitant of *Rhodophyta*).

Colonies on MA are light yellow, circular and convex. Cells are Gram-stain-negative, strictly aerobic, and non-motile short rods. Growth occurs at 15–25 °C (optimum, 25 °C) and pH 6.0–8.0 (optimum, 7.0), and in the presence of 0.5–2.5% (w/v) NaCl (optimum, 1.5%). Oxidase and catalase activities are positive. Nitrate is not reduced to nitrite. Aesculin and gelatin are hydrolysed, but casein, l-tyrosine, Tween 20, Tween 80 and starch are not. Glucose fermentation and indole production are negative. *β*-Galactosidase and urease activities are positive, but arginine dihydrolase activity is negative. Assimilation of d-glucose, d-mannose, *N*-acetylglucosamine, d-maltose, malic acid, trisodium citrate, l-arabinose, d-mannitol, capric acid, phenylacetic acid, potassium gluconate and adipic acid is negative. Q-10 is the sole respiratory quinone, and PG, PC, an unidentified amino lipid and an unidentified amino lipid are detected as major polar lipids. The major fatty acids (>5 % of the total fatty acids) are C_18 : 0_* ω*7*c*, C_18 : 0_* ω*7*c* 11-methyl, C_16 : 0_ and C_15 : 0_ 2-OH.

The type strain is SS1-5^T^ (=KACC 22649^T^=JCM 35753^T^), isolated from red marine algae (*B. catenata*) collected from the coast of Sinsi island, Republic of Korea. The genome size and DNA G+C content of the type strain are 4472 kb and 58.3% (calculated from the whole-genome sequence), respectively. The GenBank accession numbers for the 16S rRNA gene and genome sequences of strain SS1-5^T^ are OM760815 and CP151764 and CP151767, respectively.

## Description of *Yoonia phaeophyticola* sp. nov.

*Yoonia phaeophyticola* (phae.o.phy.ti′co.la. N.L. neut. pl. n. *Phaeophyta*, the division of the brown algae; L. suffix. -*cola* (from L. masc. or fem. n. *incola*), inhabitant, dweller; N.L. fem. n. *phaeophyticola*, inhabitant of *Phaeophyta*).

Colonies on MA are light yellow, circular and convex. Cells are Gram-stain-negative, strictly aerobic and non-motile short rods. Growth occurs at 15–30 °C (optimum, 25 °C) and pH 6.0–8.0 (optimum, 7.0) and in the presence of 0.5–2.0% (w/v) NaCl (optimum, 1.5%). Oxidase and catalase activities are positive. Nitrate is not reduced to nitrite. Aesculin and gelatin are hydrolysed, but casein, l-tyrosine, Tween 20, Tween 80 and starch are not. Glucose fermentation and indole production are negative. *β*-Galactosidase and urease activities are positive, but arginine dihydrolase activity is negative. Assimilation of d-glucose, d-mannose, *N*-acetylglucosamine, d-maltose, malic acid, trisodium citrate, l-arabinose, d-mannitol, capric acid, phenylacetic acid, potassium gluconate and adipic acid is negative. Q-10 is the sole respiratory quinone, and PG, PC and an unidentified amino lipid are detected as major polar lipids. The major fatty acids (>5% of the total fatty acids) are C_18 : 0_* ω*7*c* and C_16 : 0_.

The type strain is BS5-3^T^ (=KACC 22648^T^=JCM 35751^T^), isolated from brown marine algae (*Sargassum* sp.) collected from the coast of Byeonsan, Republic of Korea. The genome size and DNA G+C content of the type strain are 3982 kb and 56.4% (calculated from the whole-genome sequence), respectively. The GenBank accession numbers for the 16S rRNA gene and genome sequences of strain BS5-3^T^ are OM760806 and CP150951–CP150953, respectively.

## supplementary material

10.1099/ijsem.0.006545 Fig. S1.

## Abbreviations

ANI: average nucleotide identity
ASW: artificial seawater
CAZyme: Carbohydrate-Active enZyme
dDDH: digital DNA–DNA hybridization
DPG: diphosphatidylglycerol
MA: marine agar
MB: marine broth
ML: maximum-likelihood
MP: maximum-parsimony
NJ: neighbour-joining
PC: phosphatidylcholine
PE: phosphatidylethanolamine
PG: phosphatidylglycerol
Q-10: ubiquinone-10
