# Supplementary material for: Yoonia algicola sp. nov., Yoonia rhodophyticola sp. nov. and Yoonia phaeophyticola sp. nov., isolated from marine algae
Source: Int J Syst Evol Microbiol. 2024 Oct 16;74(10):006545. doi: 10.1099/ijsem.0.006545 (PMC11482539; doi:10.1099/ijsem.0.006545)
Supplement: Fig. S1. [file ijsem-74-06545-s001.pdf]

Supplementary Information

Fig. S1. Phylogenetic trees based on 16S rRNA gene sequences with maximum-likelihood (a) and maximum-parsimony (b) algorithms showing the phylogenetic relationships between strains G8-12^T, SS1-5^T, and BS5-3^T and closely related taxa. Bootstrap values above 70% are indicated at the nodes as percentages from 1000 replicates. *Stappia stellulata* NBRC 15764^T (AB680962) served as the outgroup. The scale bars in the ML and MP trees represent nucleotide changes per nucleotide positions and nucleotide changes over the whole 16S rRNA sequences, respectively.

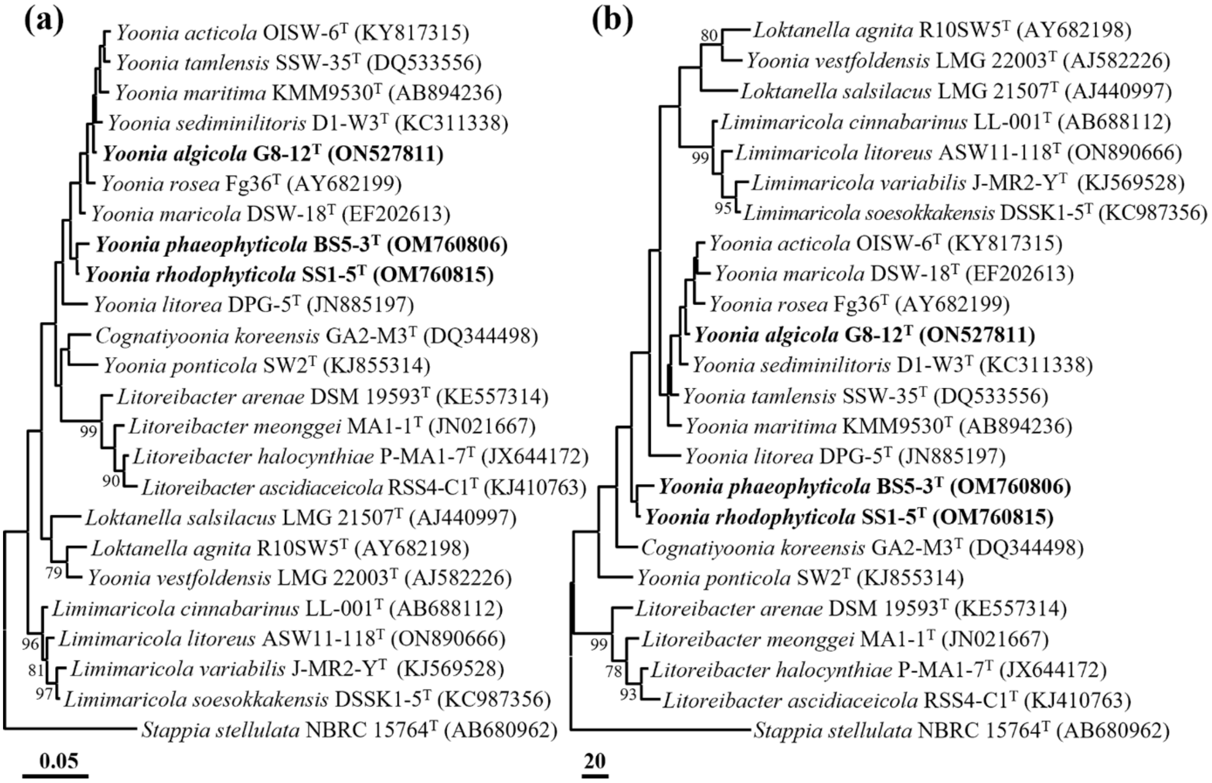

Fig. S2. Transmission electron micrographs of negatively stained (using 2% (w/v) uranyl acetate) cells showing the general morphologies of strains G8-12^T (a), SS1-5^T (b), and BS5-3^T (c) grown on marine agar for 3 days at 25°C. Scale bars, 0.5 μm.

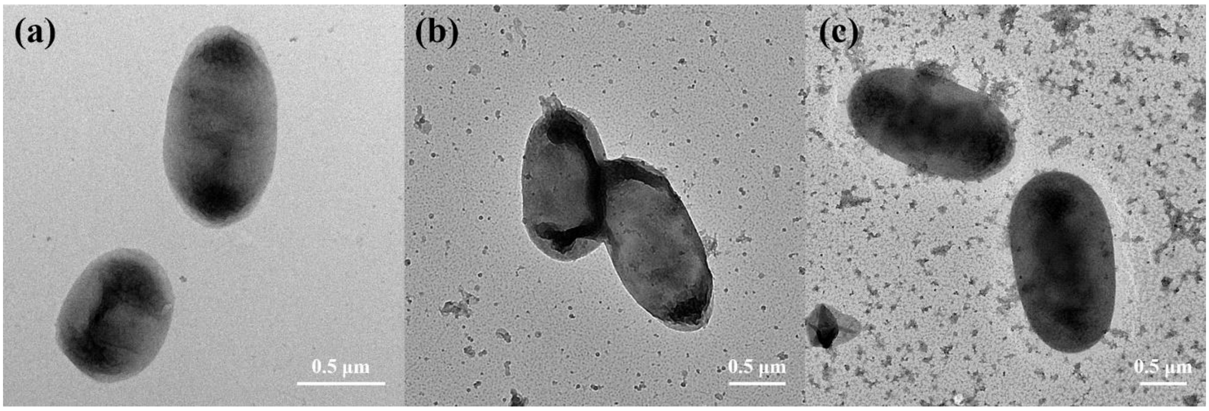

Fig. S3. Two-dimensional thin-layer chromatograms (TLC) showing the total polar lipids of strains G8-12^{T}, SS1-5^{T}, and BS5-3^{T} and *Y. vestfoldensis* KACC 13739^{T}. Solvent systems: (I) chloroform-methanol-water (65:25:4, v/v/v) and (II) chloroform-acetic acid-methanol-water (80:15:12:4, v/v/v/v). The TLC plates were sprayed with 10% ethanolic molybdophosphoric acid (a), ninhydrin (b), Dittmer-Lester (c), and Dragendorff (d) reagents for the detection of total polar lipids, aminolipids, phospholipids, and phosphatidylcholine, respectively. Abbreviations: PG, phosphatidylglycerol; PC, phosphatidylcholine; DPG, diphosphatidylglycerol; AL, unidentified aminolipid; L, unidentified lipid.

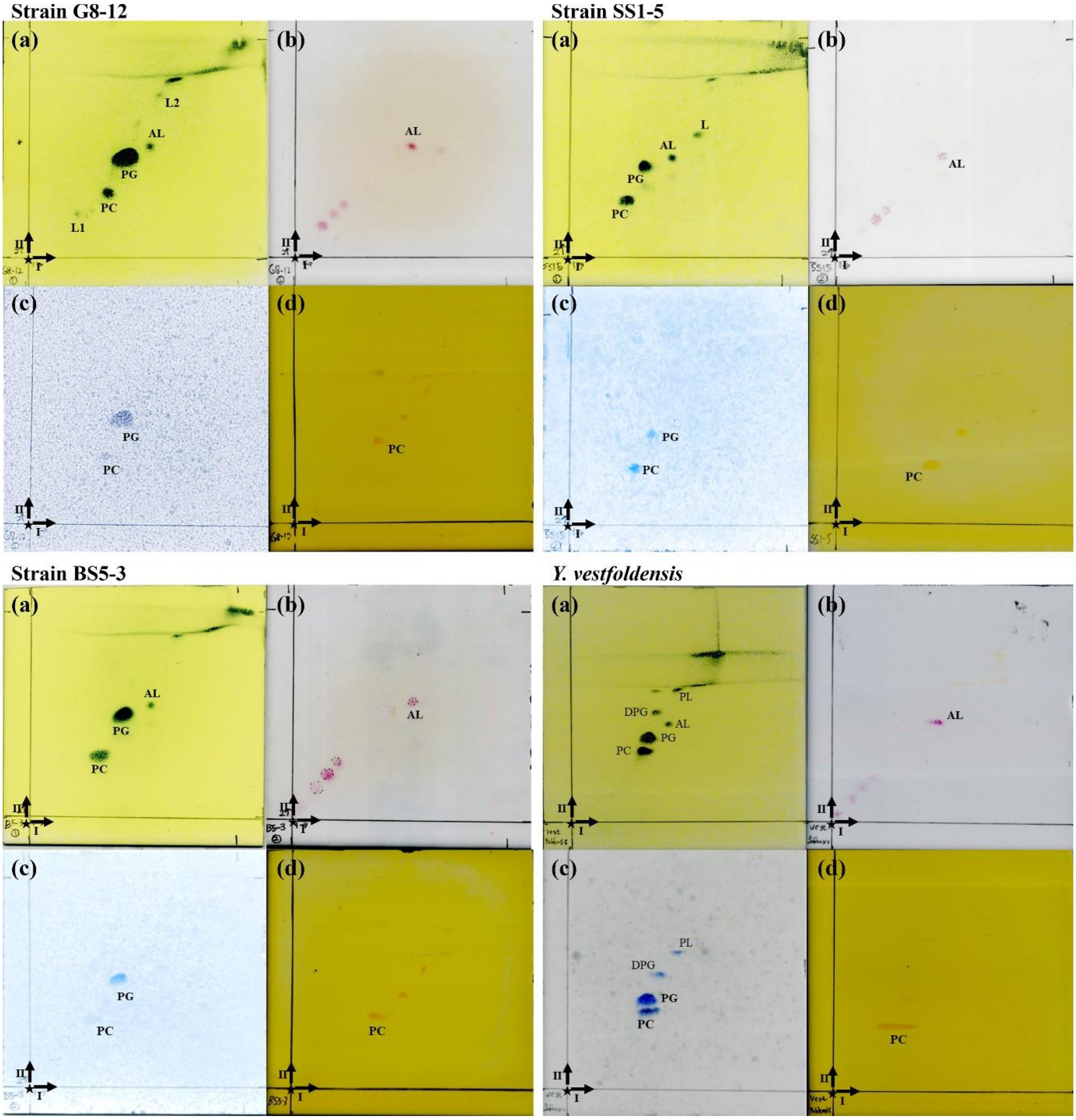

Table S1. Ecological distribution of strains G8-12^T, SS1-5^T, and BS5-3^T by searching their 16S rRNA gene sequences against metagenomic 16S rRNA amplicon datasets using the IMNGS platform, with a 99.0% sequence similarity threshold. 'Matched no.' refers to the number of metagenome datasets containing sequences that match the 16S rRNA gene sequence of the strains, while 'ARA' refers to the average relative abundance of these matched sequences within the datasets. Results where ARA was less than 0.1% for all strains were not included.

Metagenome datasets	No. of datasets	Strain G8-12^T		Strain SS1-5^T		Strain BS5-3^T	
		Matched no.	ARA (%)	Matched no.	ARA (%)	Matched no.	ARA (%)
Marine metagenome	37438	363	3.67	595	0.88	125	0.23
Aquatic metagenome	10493	226	0.65	53	1.02	10	0.06
Seawater metagenome	3225	32	0.80	163	0.78	11	0.01
Sediment metagenome	4605	150	0.41	14	0.21	3	0.05
Oyster metagenome	319	4	0.66	95	2.44	0	0
Coral metagenome	2795	15	0.07	78	0.43	33	0.29
Marine sediment metagenome	3244	62	0.29	22	0.09	1	0.31
Plant metagenome	12101	12	0.02	36	0.06	2	1
Metagenome	18630	25	0.20	31	0.09	3	0.05
Tripneustes gratilla	26	11	0.19	22	0.15	15	0.12
Beach sand metagenome	180	5	0.02	22	0.40	0	0
Gut metagenome	42163	14	0.09	20	0.29	2	0.001
Estuary metagenome	413	17	0.08	8	0.13	0	0
Sponge metagenome	653	0	0	14	0.26	2	0.003
Marine plankton metagenome	151	0	0	14	0.91	0	0
Microbial mat metagenome	720	13	0.06	3	0.21	1	0.01
Biofilm metagenome	2953	12	0.54	4	0.75	0	0
Air metagenome	1047	1	0.002	11	0.34	0	0
Triticum aestivum	886	0	0	7	0.16	0	0
Bioreactor metagenome	2376	1	0.002	5	0.24	1	0.47
Bacterium	994	5	0.15	1	0.22	0	0
Unidentified	877	0	0	5	2.03	0	0
Uncultured bacterium	4127	4	0.07	3	0.68	2	0.08
Algae metagenome	689	3	0.20	4	0.04	0	0
Fish gut metagenome	1000	3	0.02	4	0.12	0	0
Rhizosphere metagenome	14155	3	0.10	4	0.01	0	0
Seminavis robusta	9	1	0.01	3	0.45	3	0.29
Amplicon sequences	33	3	0.73	0	0	0	0
Metagenomes	1568	3	0.23	0	0	0	0
Plastic metagenome	14	3	0.87	0	0	0	0
Salt marsh metagenome	22	3	0.37	0	0	0	0
Mollusc metagenome	157	0	0	3	0.14	0	0
Rock metagenome	169	0	0	3	0.12	0	0
Invertebrate metagenome	43	1	0.02	1	0.28	2	0.03
Annelid metagenome	37	0	0	2	0.16	1	0.001
Epibiont metagenome	132	0	0	2	1.38	2	0.01
Lake water metagenome	621	2	0.12	0	0	0	0
Hypersaline lake metagenome	57	1	0.40	0	0	0	0
Mouse gut metagenome	19703	1	0.44	0	0	0	0
Salt lake metagenome	11	1	4.94	0	0	0	0
Freshwater sediment metagenome	1494	0	0	1	0.11	0	0
Rat gut metagenome	1422	0	0	1	0.18	0	0
Capasa incensata	1	0	0	0	0	1	0.49
Panulirus ornatus	20	0	0	0	0	1	0.47

Table S2. Genome relatedness among strains G8-12^T, SS1-5^T, and BS5-3^T and closely related type strains of the genus *Yoonia*

Taxa: 1, strain G8-12^T (CP151762–3); 2, strain SS1-5^T (CP151764, CP151767); 3, strain BS5-3^T (CP150951–3); 4, *Y. maricola* DSM 29128^T (PGTY00000000); 5, *Y. rosea* DSM 29591^T (FTPR00000000); 6, *Y. sediminilitoris* DSM 29955^T (QBUD00000000); 7, *Y. vestfoldensis* DSM 16212^T (ARNL00000000).

		dDDH† value (%)						
		**1**	**2**	**3**	**4**	**5**	**6**	**7**
ANI† value (%)	**1**	–	19.5	19.2	21.1	26.9	19.5	18.8
	**2**	74.6	–	20.1	19.5	19.2	19.1	18.9
	**3**	74.8	74.5	–	19.5	19.4	19.4	19.1
	**4**	78.3	74.3	74.6	–	20.6	19.1	18.7
	**5**	83.5	74.6	74.8	78.2	–	19.6	19.3
	**6**	74.6	74.8	73.8	74.1	74.4	–	19.1
	**7**	75.2	74.4	73.9	74.2	75.1	74.2	–

†ANI, average nucleotide identity; dDDH, digital DNA-DNA hybridization.

Table S3. Comparison of cellular fatty acid compositions (%) of strains G8-12^T, SS1-5^T, and BS5-3^T and closely related type strains of the genus *Yoonia*

Taxa: 1, strain G8-12^T; 2, strain SS1-5^T; 3, strain BS5-3^T; 4, *Y. maricola* KCTC 12863^T; 5, *Y. rosea* KCTC 22197^T; 6, *Y. sediminilitoris* KCTC 32383^T; 7, *Y. vestfoldensis* KACC 13739^T. All data were obtained from this study. Data are expressed as percentages of the total fatty acids, and fatty acids constituting less than 1.0% in all strains are not shown. Major components (>5.0%) are highlighted in bold. tr, trace amount (<1.0%); –, not detected.

**Fatty acid**	**1**	**2**	**3**	**4**	**5**	**6**	**7**
Saturated:							
$C_{12:0}$	tr	1.5	tr	tr	tr	tr	tr
$C_{14:0}$	tr	1.3	tr	tr	1.3	tr	tr
$C_{16:0}$	**7.0**	**12.3**	**6.6**	**6.7**	**14.3**	3.9	**7.8**
$C_{17:0}$	tr	3.1	tr	1.2	2.0	tr	1.3
$C_{18:0}$	2.4	1.4	3.6	2.8	**8.9**	3.1	2.8
Unsaturated:							
$C_{18:1}$ $\omega7c$	**74.7**	**46.4**	**80.4**	**69.5**	**55.2**	**71.7**	**65.9**
$C_{18:1}$ $\omega7c$ 11-methyl	4.5	**13.7**	tr	**10.1**	4.4	1.7	**5.4**
Hydroxy:							
$C_{10:0}$ 3-OH	2.7	–	2.8	tr	2.0	2.7	3.5
$C_{12:1}$ 3-OH	2.5	4.1	2.8	2.5	3.4	4.4	3.1
$C_{15:0}$ 2-OH	–	**6.5**	tr	tr	tr	–	tr
iso-$C_{12:0}$ 3-OH	–	tr	tr	tr	tr	tr	1.3
Summed feature*:							
3 ($C_{16:1}$ $\omega6c$ and/or $C_{16:1}$ $\omega7c$)	tr	2.4	tr	tr	1.2	tr	tr
6 ($C_{19:1}$ $\omega9c$ and/or $C_{19:1}$ $\omega11c$)	tr	2.4	tr	tr	tr	–	–

*Summed features are groups of fatty acids that cannot be resolved reliably using the chromatographic conditions chosen. The MIDI system groups these fatty acids together as one feature with a single percentage of the total.
